# SkinAxis: 3D force-sensing soft robotic skins balancing touch sensitivity and impact protection for safe pHRC

**DOI:** 10.3389/frobt.2026.1937934

**Published:** 2026-09-23

**Authors:** Pasquale Ferrentino, Milan Amighi, Sadegh Zouelm, Théo Le Signor, Joost Brancart, Constantin Scholz, Jeroen Didden, Jelle Vanolst, Gaël Close, Bram Vanderborght

**Affiliations:** 1 Brubotics, Vrije Universiteit Brussel and IMEC, Brussels, Belgium; 2 Melexis Technologies SA, Bevaix, Switzerland; 3 Sustainable Materials Engineering (SUME), Vrije Universiteit Brussel, Brussels, Belgium; 4 Melexis Technologies NV, Ypres, Belgium

**Keywords:** finite element analysis (FEA), impact safety (ISO/TS 15066), physical human-robot collaboration, soft robotic skin, tactile force sensing

## Abstract

Physical human-robot collaboration (pHRC) requires robotic skins that simultaneously ensure impact safety, as established by ISO/TS 15066, and preserve touch sensitivity for delicate human interactions. Existing soft skins enhance compliance and multimodal sensing, but their design is rarely guided by a quantitative evaluation of the trade-off between touch sensitivity and impact mitigation, and systematic impact testing against ISO/TS 15066 limits is often absent. The SkinAxis modular soft robotic skin addresses this gap by embedding a compact 3D force sensor in the soft material, directly enabling touch localization, and using a material-based design that jointly optimizes quasi-static touch sensitivity and impact safety. This purpose is achieved by combining the mechanical properties of elastomeric materials, selected through dedicated mechanical tests, to identify a stiff elastomer that efficiently transfers forces to the sensor and a highly dissipative elastomer that absorbs impact energy. To highlight the trade-off achieved by the selected materials, two main designs are benchmarked: a single-material baseline and a hybrid patch with a compliant surface, a rigid load-transmission plate, and a stiff sensor support sleeve. The designs are compared in terms of quasi-static force sensitivity $\left(k_{\varepsilon }\right)$ , touch localization performance, and maximum pressure, as well as peak-force and transmitted-energy reduction efficiencies ( $P_{\text{max}}$ , $\eta_{\text{F}}^{\left(-\right)}$ , $\eta_{\text{E}}^{\left(-\right)}$ ), measured respectively in quasi-static compression, force-indentation, and impact experiments on a human head surrogate (PILZ PRMS setup). The results demonstrate that the hybrid solution achieves higher quasi-static force sensitivity $\left(k_{\varepsilon }=0.089\right)$ , a more reliable model for touch localization; it reaches 44.66% reduction in peak force and 18.95% reduction of transmitted energy under impact at cobot’s operational velocity of 1 $\frac{m}{s}$ , with a maximum transmitted pressure of 1.11 MPa in accordance with ISO/TS 15066 limits. In parallel, finite element simulations predict the forces transmitted to the embedded force sensor in quasi-static compression and impact conditions, supporting the experimental findings.

## 1 Introduction

Robots in various embodiments, such as industrial arms, humanoids, and quadrupeds, are increasingly deployed in crowded, shared environments where they must operate quickly and productively while remaining safe in the presence of humans, animals, and obstacles. Safety in these collaborative applications is regulated internationally by international standards such as ISO/TS 15066 (International Organization for Standardization, 2011), which define limits on speed and on the forces and pressures that robot systems may apply to different parts of the human body. Meeting these limits typically requires two complementary approaches: active, control-based strategies and passive, design-based strategies. Active strategies equip the robot with external sensor systems and real-time control algorithms that make it situationally intelligent, enabling it to adapt its motion in response to static and dynamic obstacles, thereby completely avoiding impact. The most common category is collision avoidance, in which collaborative applications must slow down or stop when an obstacle (i.e., a human) is detected, relying on innovative sensor technologies beyond camera systems, including radar, proximity sensors, and LiDAR (Pa et al., 2023; Morato et al., 2014; Scholz et al., 2024; Faraj and Albaker, 2025). Passive, design-based strategies aim to make robots safer by design, for example, by using sensorized protective skins that are activated by human touch and directly contribute to safe physical interaction. These protective skins mitigate impact energy and transmitted force, providing mechanical compliance across robot surfaces. They also offer distributed contact perception and enable collision detection, supporting richer physical human–robot collaboration (pHRC) (Prati et al., 2021; Pang et al., 2021). Existing approaches can be broadly categorized by the material solution adopted. TacSuit is a wearable, large-area, bioinspired tactile suit that wraps collaborative robots with modular fabric-based patches, combining soft textile layers and flexible substrates to conformally cover complex geometries and provide comfortable contact over extended surfaces (Zhou et al., 2023). CoboSkin, in contrast, is a fluid-driven soft skin in which pneumatic actuators and soft chambers are embedded in a compliant structure attached around the robot arm (Pang et al., 2020). By tuning the internal air pressure, CoboSkin can significantly vary its stiffness and cushioning behavior, thereby reducing peak collision forces and adapting the skin’s mechanical response to different tasks. Along similar lines, AIRSKIN implements a commercial solution of an adaptive pneumatic skin for collaborative robots, in which pressurized soft modules provide impact cushioning, distributed contact detection, and safety-certified protection (Rustler et al., 2024). Other works focus on soft elastomeric skins with conformal adaptability, such as the serpentine-structured robot skin (Ye et al., 2022), in which stretchable elastomeric elements interconnect to endow the skin with high conformability and close mechanical coupling with curved robot links and on-body surfaces. Beyond these solutions, lattice and cellular structures have recently been explored as mechanically programmable skins. Magnet-based soft robotic skins using 3D-printed multi-lattice structures (Bang et al., 2026) and lattice-patterned piezoresistive tactile skins (Jamshidi et al., 2024) show how cellular architectures can distribute compliance and sensing over large areas. In parallel, other contributions explore low-cost, easy-to-build soft skins based on elastomeric or foam materials with embedded pressure-sensitive components that can be mounted on standard collaborative manipulators to democratize tactile safety in industrial settings (Park et al., 2024). Multifunctional electronic skins further integrate proximity, pressure, temperature, and wide-range contact-force sensing with low channel coupling, enabling obstacle avoidance, safe physical guidance, and human-in-the-loop teaching in complex pHRC scenarios (Ge et al., 2023; Li et al., 2022).

Despite this progress, the design of robotic skins is guided by a qualitative evaluation of how material choice affects both touch sensitivity and impact mitigation. Most works emphasize sensing modalities and touch localization, and use soft materials primarily to guarantee safe touch interactions between robots and humans, but provide only limited analysis of cushioning and of how much force is transmitted through the skin to the underlying robot or to a human body part as a function of material stiffness, damping, and thickness. In particular, systematic characterization of impact conditions for controlled operational velocities-such as energies and resulting force-time profiles- and explicit comparison against ISO/TS 15066 limits on force and pressure are often absent, even though they are increasingly needed to integrate collaborative robotic systems into workspaces that demand high productivity at higher operational velocities. As a result, it remains unclear how to select and combine soft materials in robotic skins to achieve localized touch perception while simultaneously ensuring impact safety in deployment-ready collaborative applications.

This work proposes SkinAxis, shown in Figure 1, a modular soft robotic skin for collaborative applications. The SkinAxis patch consists of a soft protective layer mechanically coupled to a compact three-dimensional force sensor (Tactaxis®, developed by Melexis). The Tactaxis® sensor uses a multi-pixel gradiometric Hall-effect architecture that suppresses spatially uniform magnetic stray fields through differential measurement, while retaining the local field gradients generated by its embedded permanent magnet (Le Signor et al., 2022; Le Signor et al., 2023). Accordingly, no additional passive magnetic shielding was used in the present patch integration. The SkinAxis design combines elastomeric layers, a rigid load-transmission plate, and a stiff sensor support sleeve to jointly enhance touch sensitivity and mitigate dynamic impact loads, thereby complying with ISO/TS 15066 while remaining comfortable to the touch. To this end, elastomeric materials are systematically selected to identify a stiff elastomer that efficiently transmits load to the sensor and a highly dissipative elastomer that absorbs impact energy. These materials are then combined in two main designs-a single-material baseline and a hybrid patch with a compliant encapsulation, a rigid load-transmission plate, and a stiff sensor support sleeve-which highlight the fundamental trade-off between touch sensitivity and impact mitigation and provide quantitative metrics for safe, tactile skins applicable to collaborative robotic arms, humanoids, and quadrupeds. The two designs are evaluated in terms of quasi-static force sensitivity, touch localization performance, and impact safety indices based on maximum transmitted pressure, peak impact force, and transmitted energy. These quantities were measured using quasi-static compression, force-indentation, and controlled-collision experiments on a human head surrogate. These metrics reveal that the hybrid design achieves higher force sensitivity, more reliable touch localization, and substantial reductions in peak force and transmitted energy under impact, in accordance with ISO/TS 15066 limits.

**FIGURE 1:**
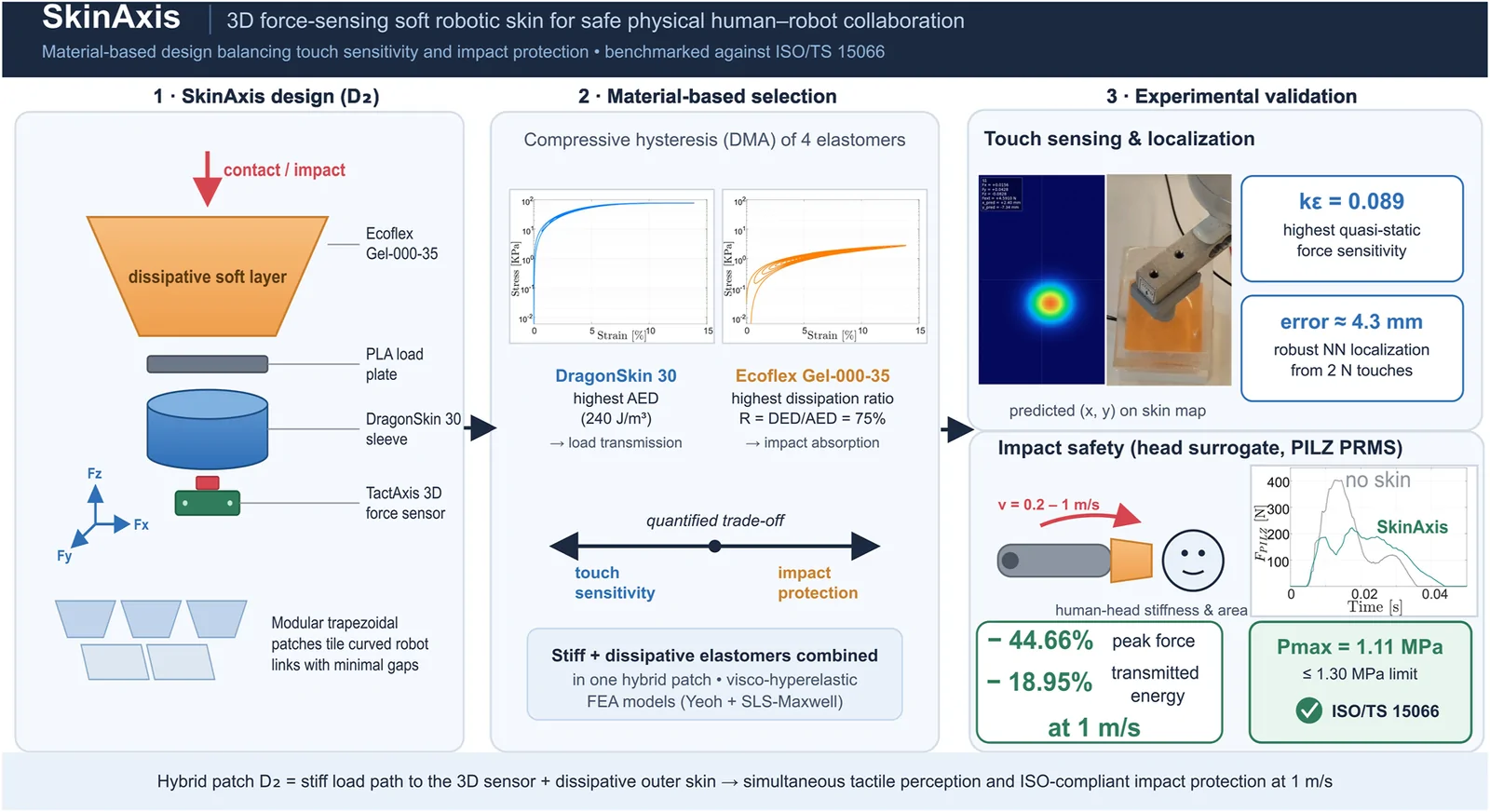
Graphical abstract of the paper.

This paper is organized as follows: Section 2.1 describes the reference SkinAxis design and its underlying material-based concept, as well as the baseline design used for benchmarking. Section 2.2 then presents the material characterization methodology and the absorbed and dissipated energy metrics used to select the elastomers adopted in the designs. Sections 2.3, 2.4, and Section 2.5 describe the experimental and numerical protocols for quasi-static compression, touch localization, and impact tests on the human head surrogate. Section 3 reports the main results on quasi-static force sensitivity, touch localization performance, and impact safety, highlighting the trade-off between the single-material and hybrid designs. Finally, Section 4 discusses the implications of these findings and outlines directions for future work on deployment-ready tactile skins for robotics applications.

## 2 Materials and methods

In the following subsections, the methodology for selecting materials for the SkinAxis patch design and the metrics used to assess touch sensitivity and impact mitigation will be described.

### 2.1 Skin design

Two skin designs, denoted D_1_ and D_2_, are compared to systematically explore the fundamental trade-off between impact energy absorption and touch sensitivity. These designs serve as proof-of-concept implementations of the material-selection methodology described later in Section 2.2, and are used to quantify how combining stiff and dissipative elastomers influences 3D sensor performance under both quasi-static and dynamic loading conditions. Both patches are fabricated by molding soft elastomeric layers around the embedded force sensor.

- D_1_ serves as the single-material baseline. It features a trapezoidal soft skin layer with height H=24 mm, upper base dimensions of 65×35 mm, and a lower base of 35×35 mm. The trapezoidal geometry is chosen to allow multiple SkinAxis patches to be tiled along curved robot links with minimal interstitial gaps (as shown in Figure 2b), thereby ensuring continuous protection and sensing coverage rather than leaving unprotected regions between adjacent modules. The 3D force sensor is directly embedded in a dedicated cavity at the bottom of the trapezoidal skin, which is formed in the elastomer during molding, ensuring tight mechanical coupling between the soft skin and the sensing element. Two variants of D_1_ are tested: one based on the dissipative gel elastomer, labeled D_1_-gel, and another based on the stiffer and more elastic elastomer, labeled D_1_-dragon. This baseline therefore provides a direct comparison between damping-oriented and load-transmitting behavior in a single-material skin (Figure 2a, left).
- D_2_, in contrast, adopts a hybrid approach to combine dissipative and stiff material properties within a single patch. This design incorporates a thinner, soft layer ( H=17 mm; base 40×27 mm), made of the dissipative elastomeric gel, coupled to the sensor via a 4 mm-thick PLA load-transmission plate that efficiently channels surface deformations. The sensor resides within a 33 mm-diameter × 7 mm-high support sleeve, manufactured from a stiff and elastic elastomer. The sleeve provides a mechanically robust housing for the sensor while acting as a compliant, high-gain interface that converts local deformations of the surrounding soft structure into force variations at the sensing element. This configuration is designed to ensure stable and repeatable force transmission under both quasi-static interactions and high-speed impacts, while still providing a soft, dissipative outer skin (Figure 2a, right).

**FIGURE 2:**
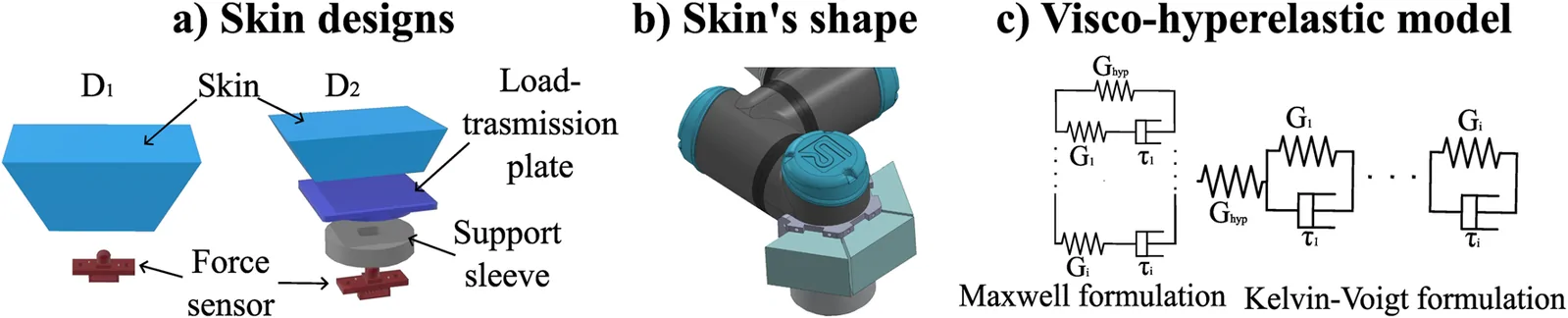
**(a)** The two skin designs ( ${\text{D}}_{1}$ , ${\text{D}}_{2}$ ) compared in this work were assessed for touch sensitivity and impact force mitigation performance. **(b)** The trapezoidal shape of the skin is justified to allow multiple patches to be positioned along curved robot links with minimal interstitial gaps. **(c)** General formulations of visco-hyperelastic constitutive models are used in this work. Both feature a hyperelastic spring $\left(G_{\mathrm{hyp}}\right)$ either in parallel with a Maxwell viscoelastic branch (right) or in series with Kelvin-Voigt branches (left).

Throughout the paper, D_1_-gel and D_1_-dragon are used as single-material baselines to expose two extremes of the trade-off between touch sensitivity and impact mitigation, whereas D_2_ is adopted as the reference SkinAxis design in this study, which attempts to balance these objectives. The three patches are assessed through simulations and validation experiments to measure their quasi-static force sensitivity, touch localization performance, and impact safety in controlled collision scenarios.

### 2.2 Material selection

The first step in the analysis is selecting the skin material. In this section, a general metric is defined to classify materials based on the trade-off between load transmission and impact mitigation, a central tenet of SkinAxis’s design. For safety under impacts, the skin must be soft and strongly dissipative, so that impact energy and peak forces are attenuated before reaching the human body. For touch sensitivity, however, the skin must still transmit sufficient load to the embedded force sensor so that small contact forces remain measurable and localized. The material selection methodology is therefore designed to quantify this apparent contradiction between safety and sensing and to provide a principled way to choose and combine elastomers that balance these objectives. Several elastomers are considered, covering a wide range of stiffnesses and damping properties; they are tested using compression amplitude-sweep tests, with a logarithmic increase in strain amplitude from 0.1% to 14% (compression taken as positive by convention) at a fixed frequency of 0.1 Hz, using a Dynamic Mechanical Analyzer (DMA RSA-G2, TA Instruments). In this sense, the amplitude-sweep tests were designed as a low-rate comparative screening procedure to rank the candidate elastomers by their relative energy storage and dissipation behaviors under common loading conditions, rather than to reproduce the transient loading history of the collision experiments. Cylindrical specimens have a radius of r=10 mm and a height of H=20 mm. Materials are ranked using two key quantities:

- Absorbed Energy Density (AED): energy absorbed per unit volume, calculated by integrating the loading phase of the stress–strain curve.
- Dissipated Energy Density (DED): energy dissipated per unit volume, obtained as the difference between the loading- and unloading-phase integrals.

In this study, the energy-based metric was adopted to characterize the mechanical response of the investigated skin materials for two main reasons. First, the tested materials exhibit visco-hyperelastic behavior in large deformation regime; therefore, their response is nonlinear and depends on the deformation level and loading conditions. A stiffness-based descriptor, such as a single Young’s modulus evaluated over a limited strain range, cannot fully represent the mechanical behavior over the finite deformation range relevant to the considered contact scenario. Second, an energy-based representation is more direct and physically meaningful in the context of impact safety. The energy absorbed during loading and the energy dissipated during unloading are directly related to the ability of the skin patch to attenuate an impact and transmit the energy to the underlying sensing element and, ultimately, to the human body. Accordingly, the absorbed energy density was used as a task-oriented metric to compare the skin patches under the same experimental loading conditions.

In the context of SkinAxis, AED refers to the material’s ability to store and transmit mechanical energy within the skin structure, thereby enhancing the embedded force sensor’s sensing performance. The DED alone is insufficient to compare materials for impact mitigation, because it scales with the total absorbed energy (AED) and can therefore be large even for stiff, weakly dissipative elastomers. For collision safety, a more appropriate metric is the energy dissipation ratio $R=\frac{DED}{AED}$ , which normalizes dissipation by the absorbed energy and measures how efficiently a material converts mechanical work into damping. High R thus identifies materials that effectively attenuate impact forces. Hence, two materials are selected for the SkinAxis patch design (Section 2.1): one exhibiting a high AED, representative of stiff, load-transmitting behavior, and one maximizing the energy dissipation ratio R , representative of strongly dissipative, protective behavior. The mechanical performance of the resulting skin patches is then benchmarked experimentally and numerically via Finite Element Analysis (FEA) under quasi-static and impact conditions (Ferrentino et al., 2023). These simulations are used to validate the forces read by the force sensor embedded in the SkinAxis patch, as detailed in Sections 2.3 and 2.5.

To model the material behavior in FEA (Ferrentino et al., 2023), the material-based methodology introduced in (Ferrentino et al., 2021; Ferrentino et al., 2022) is adopted. This approach defines the constitutive equations of the materials through dedicated mechanical characterization tests. The selected elastomers are assumed to exhibit hyperelastic and visco-hyperelastic behavior, as they must sustain large deformations and, in particular, the material that maximizes R must dissipate energy under impact loading. Accordingly, the following mechanical tests, all performed using a DMA RSA-G2, are conducted for material characterization:

- Quasi-static tensile tests on rectangular specimens ( W×H×L=4×3×30 mm) at a strain rate of 1%/s. These tests provide stress–strain curves, which are used to fit several hyperelastic models and estimate the parameters of the strain energy density function.
- Dynamic tensile frequency sweeps (0.1–10 Hz, 1% strain amplitude). These tests yield the storage $\left(G^{\prime }\right)$ and loss $\left(G^{\prime \prime }\right)$ shear moduli as functions of frequency, enabling the identification of viscoelastic parameters associated with Kelvin–Voigt or Maxwell branches.

The final visco-hyperelastic model combines hyperelastic equilibrium springs with viscoelastic branches arranged either in parallel (Maxwell) or in series (generalized Kelvin–Voigt), as illustrated in Figure 2c.

### 2.3 Quasi-static skin force sensitivity analysis

To jointly achieve reliable sensing and impact safety, SkinAxis must transmit sufficient load to the embedded force sensor during gentle contact while attenuating impact forces. The quasi-static compression experiments are therefore designed to quantify the force transmitted by each skin patch to the Tactaxis® sensor under slow, controlled loading and to compare the resulting quasi-static force sensitivity across designs. Three separate Tactaxis® sensors were used, one for each skin design. Each sensor was factory-calibrated, and the same calibration was used for all skin designs; no recalibration was performed after encapsulation.

Every skin is simulated and experimentally validated under quasi-static compression to determine the relationship between the externally applied load and the force measured by the Tactaxis® sensor, thereby estimating the skin’s quasi-static force sensitivity. In the FEA (conducted via COMSOL Multiphysics), a compressive boundary displacement $u_{\text{z}}$ , with a step of 0.5 mm, is applied to the top skin surface (marked in blue in Figure 3a). The resulting vertical compressive force $F_{\text{in}}$ on the skin surface (red, Figure 3a) and the norm of the total transmitted force $F_{s}$ (green, Figure 3a) are retrieved as outputs. In addition, the PLA load-transmission plate of the hybrid D_2_ patch is modeled as a linear elastic deformable solid, with Young’s modulus of 2.06 GPa and Poisson’s ratio of 0.44. Experimental validation is performed using a Mark-10 compression machine (Figure 3b, in magenta), which records the compressive displacement (applied in 0.5 mm increments) and the corresponding force. In parallel, the Tactaxis® sensor force is acquired by connecting it to an ESP32 board.

**FIGURE 3:**
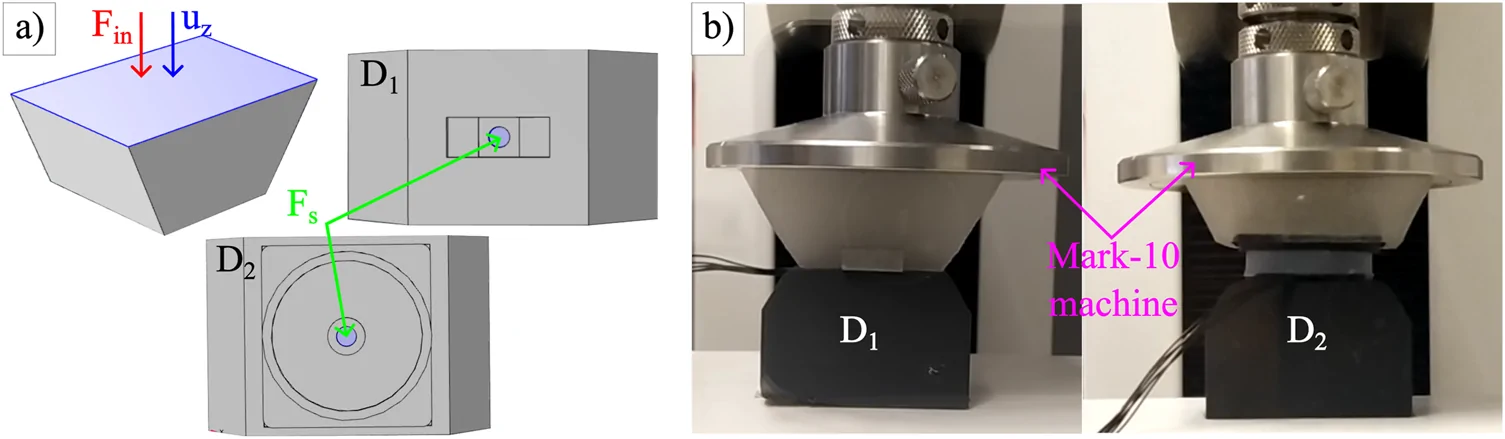
**(a)** The quasi-static simulations consist of applying a compressive load $F_{in}$ (marked in red) and measuring the compressive displacement $u_{z}$ in the two designs (labeled in blue), as well as the force norm transmitted to the sensor locations $F_{s}$ , highlighted in green for both designs. **(b)** The Mark-10 compression machine is used for the quasi-static compression validation.

Because the benchmarked skins have different thicknesses, the sensitivity is quantified using the normalized through-thickness strain $\varepsilon =\delta /H_{\text{skin}}$ , where δ is the through-thickness compression and $H_{\text{skin}}$ the initial skin thickness ( $H_{D_{1}}=24\text{ mm}$ , $H_{D_{2}}=17\text{ mm}$ ). Hence, the quasi-static force sensitivity $k_{\varepsilon }$ is defined as the slope of a linear fit relating the sensor force $F_{s}$ to the externally applied force $F_{\text{in}}$ over a common operating strain range ε∈[0,15%] :

$$F_{s}=k_{\varepsilon }F_{\text{in}}+b$$ (1)

In this formulation, $k_{\varepsilon }$ quantifies how much force is transmitted to the sensor per unit increase of externally applied force, i.e., the change in $F_{s}$ for each additional 1 N of $F_{\text{in}}$ within the chosen strain range. The term b in Equation 1 is the constant coefficient of the linear fit. The numerical values of $k_{\varepsilon }$ for D_1_-gel, D_1_-dragon, and D_2_ are reported and discussed in Section 3.2.

### 2.4 Touch localization and touch sensitivity definition

A tactile skin must also enable reliable localization of human touch on its surface. In this work, each patch integrates a compact 3D force sensor, but the touch localization capability ultimately depends on how the skin structure and materials modulate the forces transmitted to the sensor when contact occurs at different locations. These experiments are therefore designed to quantify whether and to what extent each design allows the underlying 3D force sensor to infer the point of contact on the skin, and how this performance relates to the patch’s touch sensitivity. This methodology is independent of the specific sensor type: it could equally be applied to other embedded 3D force/torque sensors, provided that the local contact forces are available as inputs. In the proposed framework, touch localization is framed as a supervised regression problem in which the model predicts the skin-frame contact coordinates $\left(x_{\text{skin}},y_{\text{skin}}\right)$ from a feature vector built from the three-axis force readings $\left(F_{x},F_{y},F_{z}\right)$ of the embedded sensor and the externally applied normal force $F_{\text{in}}$ . Specifically, the model maps the skin-frame coordinates of the contact point $\left(x_{\text{skin}},y_{\text{skin}}\right)$ on the top surface to the forces $\left(F_{x},F_{y},F_{z}\right)$ measured by the embedded Tactaxis® sensor and to $F_{\text{in}}$ . Training and validation data are collected by indenting the skin with a rigid cylindrical indenter of 10 mm diameter, indicated in red in Figure 4a, attached to the end-effector of a UR3e collaborative robot equipped with a strain-gauge force sensor (Figure 4a, in blue) that measures $F_{\text{in}}$ . For each skin patch, the robot indents the top surface at $N_{\text{loc}}=200$ distinct contact locations, randomly distributed over a sensing area of 50×25 mm to minimize boundary effects, and always including the geometric center. At every location, four different target force levels are applied. Each contact episode consists of a 3 s indentation plateau at the target force, followed by a 2 s release phase during which contact is removed before moving to the next location. Contact points were retained for model training and validation only when a stable force plateau was identified. Specifically, continuous contact had to be maintained for at least 2.5 s, during which both the input indenter force and the Tactaxis® sensor response remained approximately constant. Ramping, release, contact-loss, and transient intervals were excluded. Furthermore, a spatial location was retained only if valid plateaus were available at at least two distinct target force levels. A set of standard regression models is benchmarked for each skin design: Gaussian Process Regression with ARD squared exponential (GPR_SE) and ARD Matérn 5/2 kernels (GPR_M52), feedforward neural networks with a single hidden layer of 10 or 20 units (NN_1 × 10, NN_1 × 20) and with two hidden layers of 10 or 20 units each (NN_2 × 10, NN_2 × 20), Support Vector Regression with Gaussian kernel (SVM_Gaussian), bagged regression trees (BaggedTrees), and LSBoost regression trees (LSBoostTrees). The dataset is split by spatial point into 80% training and 20% validation sets using a fixed random seed, so that all samples from a given location belong either to the training or to the validation set. For each model, a global mean Euclidean localization error is defined over a dataset D (training or validation) as:

$$e_{\text{global}}\left(D\right)=\frac{1}{\left|D\right|}\underset{i\in D}{\sum }\sqrt{{\left(x_{\text{pred}}^{\left(i\right)}-x_{\text{skin}}^{\left(i\right)}\right)}^{2}+{\left(y_{\text{pred}}^{\left(i\right)}-y_{\text{skin}}^{\left(i\right)}\right)}^{2}}.$$ (2)

The training and validation errors are then denoted $e_{\text{train}}=e_{\text{global}}\left(D_{\text{train}}\right)$ and $e_{\text{val}}=e_{\text{global}}\left(D_{\text{val}}\right)$ , respectively. In Equation 2, $\left(x_{\text{skin}},y_{\text{skin}}\right)$ are the positions in input, while $\left(x_{\text{pred}},y_{\text{pred}}\right)$ the positions predicted by the model. the Model selection is performed primarily based on $e_{\text{val}}$ , as this quantity reflects the localization accuracy achievable on unseen data. Hyperparameters (kernel parameters, network width and depth, regularization strength, etc.) are tuned via grid search, and neural network training is monitored using the validation loss with early stopping to limit overfitting. For each skin, the configuration with the lowest validation error is retained for subsequent analysis. From the selected model for each skin, it is possible to characterize touch sensitivity as a function of indentation force by defining a force-specific mean Euclidean localization error $e_{\text{mean}}\left(F_{\text{in}},D_{\text{val}}\right)$ . This quantity is obtained by restricting the dataset to samples acquired at a given normal force level $F_{\text{in}}$ . Let $D\left(F_{\text{in}}\right)\subset D_{\text{val}}$ denote the subset of validation samples with indentation force $F_{\text{in}}$ . The corresponding mean localization error is:

$$e_{\text{mean}}\left(F_{\text{in}},D_{\text{val}}\right)=\frac{1}{\left|D\left(F_{\text{in}}\right)\right|}\underset{i\in D\left(F_{\text{in}}\right)}{\sum }\sqrt{{\left(x_{\text{pred}}^{\left(i\right)}-x_{\text{skin}}^{\left(i\right)}\right)}^{2}+{\left(y_{\text{pred}}^{\left(i\right)}-y_{\text{skin}}^{\left(i\right)}\right)}^{2}}.$$ (3)

For each skin design, Equation 3 is used to derive the index.

$$S_{\text{touch}}=\frac{e_{\text{mean}}\left(F_{\text{in}},D_{\text{val}}\right)}{F_{\min}},$$ (4)

Equation 4 is used as an indirect measure of touch sensitivity. Here, $F_{\min}$ denotes the smallest indentation force $F_{\text{in}}$ for which the mean Euclidean localization error $e_{\text{mean}}\left(F_{\text{in}},D_{\text{val}}\right)$ falls below a threshold of 10 mm in validation. For each candidate force level, the localization model is retrained using only the indentation samples corresponding to that specific force, so that $e_{\text{mean}}\left(F_{\text{in}},D_{\text{val}}\right)$ reflects the performance achievable at that force. A lower value of $S_{\text{touch}}$ therefore indicates a more sensitive skin, as it corresponds to a smaller localization error per unit of applied force.

**FIGURE 4:**
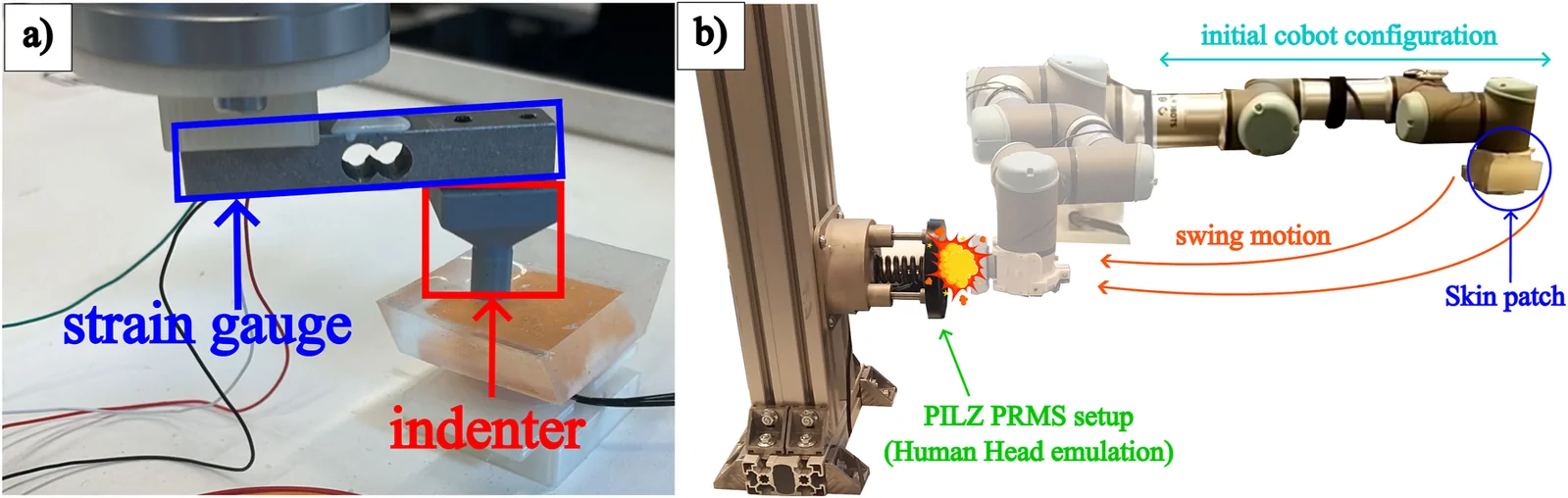
**(a)** The experimental setup for touch indentation consists of a 10 mm indenter (labeled in red) connected to the end-effector of a UR3e collaborative robot, with a strain-gauge force sensor that measures the normal force $F_{in}$ and serves as the system input. Hence, a data-driven regression model is trained for each skin type to localize touches and determine touch sensitivity. **(b)** The experimental setup for the impact tests. The UR3e cobot is positioned in a fully extended horizontal configuration and will swing and collide with the PILZ PRMS setup, which mimics human head stiffness, damping, and contact area. The cobot will first collide with the PILZ without any skin protection, then with the skin patches mounted on the end effector, to benchmark mechanical performance in terms of peak force reduction and energy transmission efficiency.

### 2.5 Impact tests for safety assessment

Skin benchmarking is also performed under impact conditions to assess impact-absorbing performance and safety warranty in human-robot physical collaboration tasks, in accordance with ISO/TS 15066.

The experiments are made using a Universal Robots UR3e in a horizontal, fully extended configuration, as shown in Figure 4b (cyan arrow; the impact direction is in the x-y plane). Every skin is mounted on the cobot end-effector (marked in blue in Figure 4b), and the robot is commanded to swing and collide with a PRMS PILZ force sensor (labeled in green in Figure 4b) at prescribed operational velocities velocities ( ${\text{v}}_{\mathrm{cobot}}$ ) of 0.2, 0.6, and 1 $\frac{m}{s}$ . The robot velocity was monitored during each trial to verify that the UR3e reached the assigned velocity before contact. The PILZ sensor is configured to emulate the stiffness and damping of the human head in accordance with the biomechanical limits of ISO/TS 15066 (International Organization for Standardization, 2011); it is circular, with a diameter of 10.8 cm, yielding a total area of 91.6 ${\text{cm}}^{2}$ . Head emulation is adopted as the worst-case scenario, given the anatomical proximity to the cervical spine and the stringent limits reported in Annex A of the ISO standard. In particular, the contact pressure limit of 1.30 MPa.

For each skin design (Section 2.1), a series of comparative impact tests is conducted and benchmarked against a baseline condition in which direct cobot-PILZ collisions occur without any skin. For each impact test, the contact force at the head surrogate (PILZ), $F_{\text{PILZ}}\left(t\right)$ , is recorded, together with the Cartesian position of the end-effector (x(t),y(t)) . The positions are projected onto the horizontal impact direction to obtain the scalar displacement s(t) , with $s\left(t_{\text{start}}\right)=0$ at the onset of contact.

The metrics that assess the protective effects of the skin patches are based on these quantities:

- The peak forces generated by the cobot-PILZ collision $\left(F_{\mathrm{peak}}\right)$ .
- The energy transmitted to the head surrogate (PILZ setup) during the impact is obtained by combining the recorded PILZ force and the projected scalar displacements in the impact direction:

$$E_{\text{trans}}=\int_{t_{\text{start}}}^{t_{\text{end}}}F_{\text{PILZ}}\left(t\right)\;ds\left(t\right).$$ (5)

In Equation 5, $t_{\text{start}}$ and $t_{\text{end}}$ are the time instants at the beginning and end of the impact.

To compare the skins against the baseline condition without skin, two relative efficiency indices are further defined based solely on quantities measured at the PILZ:

- The *relative peak-force reduction* $\left(\eta_{\text{F}}^{\left(-\right)}\right)$ :

$$\eta_{\text{F}}^{\left(-\right)}=\left(1-\frac{F_{\text{peak, skin}}}{F_{\text{peak, base}}}\right)\cdot 100,$$ (6)

In Equation 6, $F_{\text{peak, base}}$ and $F_{\text{peak, skin}}$ are the maximum contact forces at the PILZ in the baseline and skin-covered impacts, respectively.

- In addition, the *relative transmitted-energy reduction* $\left(\eta_{\text{E}}^{\left(-\right)}\right)$ based on the energy transmitted to the head:

$$\eta_{\text{E}}^{\left(-\right)}=\left(1-\frac{E_{\text{trans, skin}}}{E_{\text{trans, base}}}\right)\cdot 100,$$ (7)

In Equation 7, $E_{\text{trans, base}}$ and $E_{\text{trans, skin}}$ are the energies transmitted to the PILZ setup in baseline and skin-covered impacts, respectively.

Hence, the relative indices $\eta_{\text{F}}^{\left(-\right)}$ and $\eta_{\text{E}}^{\left(-\right)}$ quantify, respectively, the reductions in peak force and transmitted energy achieved by the skins with respect to the unprotected baseline.

During the frontal impact tests, the skin patch was centrally aligned with the PILZ PRMS and contacted it approximately normally. The nominal frontal area of the skin was 65 × 35 mm (22.75 ${\text{cm}}^{2}$ ). For pressure estimation, an effective contact area of 20 ${\text{cm}}^{2}$ was conservatively adopted, given that the full nominal skin surface may not be engaged simultaneously during a dynamic impact. The maximum transmitted pressure was calculated as the measured peak force divided by this effective contact area.

In addition, to quantify the difference between the quasi-static and impact regimes, the average nominal through-thickness strain rate was estimated as:

$${\dot{\varepsilon }}_{\text{nom,avg}}=\frac{\delta_{\max}}{H_{\text{skin}}\left(t_{\text{end}}-t_{\mathrm{start}}\right)},$$ (8)

In Equation 8, $\delta_{\max}$ is the measured peak patch compression, $H_{\text{skin}}$ is always the undeformed patch thickness, and $\left(t_{\text{end}}-t_{\text{start}}\right)$ is the measured contact duration. They are nominal patch-level estimates and do not represent local strain-rate fields within the heterogeneous patch structures, such as the D_2_ design.

Hence, for all impact trials, each patch was mounted in a single controlled configuration, centered on the end-effector and aligned with the impact direction. The PILZ head surrogate contacted the central region of the outer patch surface under nominally normal incidence. This controlled placement was maintained across skin designs and repeated trials to ensure a consistent comparison of $\eta_{\text{F}}^{\left(-\right)}$ and $\eta_{\text{E}}^{\left(-\right)}$ . The sensitivity of these metrics to lateral patch offsets, rotations, off-center contact, and oblique impacts was not investigated and remains the subject of future work. Furthermore, finite-element impact simulations were performed for each patch to model the Tactaxis® sensor force readings $\left(F_{s}\right)$ during collision. The simulations were driven by a simplified representation of the collision displacement (u(t)) , prescribed as a Gaussian-shaped pulse whose peak amplitude and duration were fitted to the experimentally measured displacement signals. The Gaussian pulse approximation respects the following equation:

$$u\left(t\right)=A\;\exp\left(\frac{{\left(t-t_{\mathrm{start}}\right)}^{2}}{2\sigma^{2}}\right),$$ (9)

In Equation 9, A is the coefficient that indicates the peak displacement during the impact and σ is the *standard deviation* of the Gaussian pulse measured through Equation 10:

$$\sigma =\frac{t_{\mathrm{end}}-t_{\mathrm{start}}}{6},$$ (10)

so that the interval $\left[t_{\mathrm{start}}-3\sigma ,\;t_{\mathrm{start}}+3\sigma \right]$ (approximately 99.7% of the pulse energy for a Gaussian profile) matches the effective contact duration observed in the experiments. In the simulation, $F_{s}$ is retrieved from the same regions explained in Section 2.3 and indicated in Figure 3A.

## 3 Results

### 3.1 Material selection results

#### 3.1.1 Material classification

The metric defined for material selection in Section 2.2 is applied to four elastomers: three from the Ecoflex series (Ecoflex Gel-000-35, Ecoflex 00-10, Ecoflex 00–30) and DragonSkin 30 (all from Smooth-On Inc., USA). These elastomers are chosen because, taken together, they span a wide range of mechanical behaviors in terms of absorbed and dissipated energy densities. Hence, they capture the fundamental trade-off between load transmission (high AED) and energy dissipation (high DED and high R=DED/AED ) that SkinAxis aims to exploit and quantify in the subsequent experimental validation of the skin designs. The mechanical behaviors are visible in Figures 5a,b, which show hysteresis loops from compressive amplitude sweeps (compression is defined as positive by convention), plotted with linear and logarithmic stress scales, respectively, for clarity. The four elastomers are marked with the following colors: Ecoflex Gel-000-35 (red), Ecoflex-00-10 (green), Ecoflex-00-30 (blue), and DragonSkin 30 (magenta). From the hysteresis loops, the Absorbed Energy Density (AED) and Dissipated Energy Density (DED) are computed for each cycle, averaged across all cycles, and reported in Figures 5c,d. As shown in Figures 5a–c, DragonSkin 30 exhibits the highest AED (240 $\frac{J}{m^{3}}$ ) and reaches higher stress levels at a given strain amplitude. Consequently, it transmits more mechanical work through the specimen. This increased load transmission amplifies the force variation at the embedded sensor for a given indentation, making DragonSkin 30 a suitable material to improve the touch sensitivity of the SkinAxis patch. In contrast, Figure 5d shows that Ecoflex Gel-000–35 has the highest energy dissipation ratio ( $R=\frac{DED}{AED}=75$ % ), indicating that it dissipates the largest fraction of the absorbed energy and is therefore well suited to endow the SkinAxis patch with impact energy dissipation properties (i.e., increased safety under impact conditions). Based on these results, DragonSkin 30 and Ecoflex Gel-000-35 are selected for the SkinAxis design.

**FIGURE 5:**
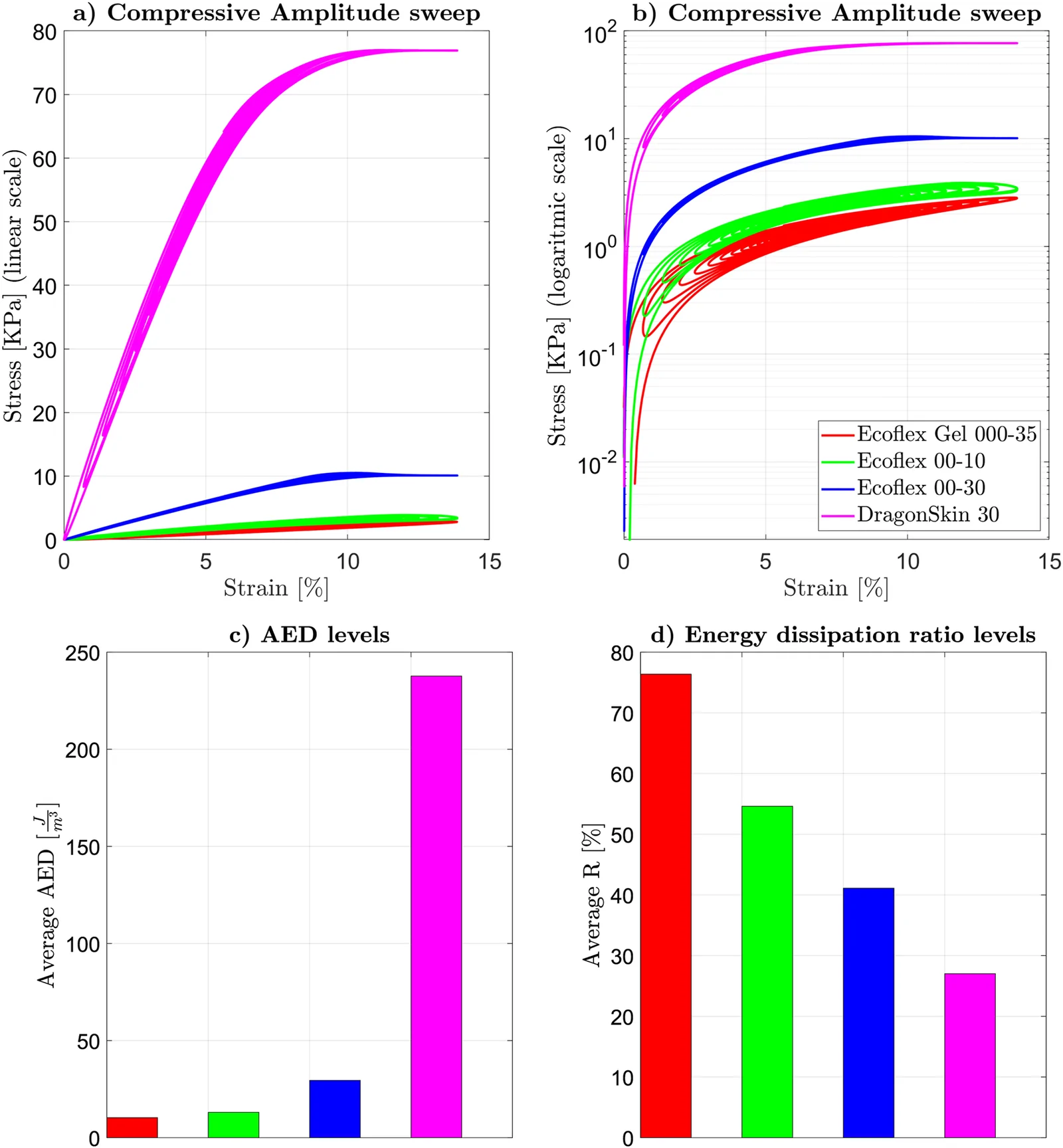
Material classification results: **(a,b)** Hysteresis loops from compressive amplitude sweeps (compression defined as positive by convention), shown with linear **(a)** and logarithmic **(b)** stress scales. Tests are performed on a DMA RSA-G2 by varying the strain amplitude logarithmically while keeping the strain frequency fixed at 0.1 Hz. Four elastomers are characterized: Ecoflex Gel-000-35 (red), Ecoflex-00-10 (green), Ecoflex-00-30 (blue), and DragonSkin 30 (magenta). **(c)** For each material, the Absorbed Energy Density (AED) is obtained as the mean area under the loading branch of all cycles. DragonSkin 30 exhibits the highest AED, indicating the greatest load-transmission capacity, which is exploited to enhance the touch sensitivity of the skin patches. **(d)** From the same tests, the Dissipated Energy Density (DED) is computed as the area enclosed by each hysteresis loop. The energy dissipation ratio $R=\frac{DED}{AED}$ is then reported, representing the fraction of the absorbed energy density that is dissipated per cycle. Ecoflex Gel-000-35 exhibits the highest R and is therefore identified as the most dissipative material for impact energy dissipation.

#### 3.1.2 Material characterization for FEA simulation

Before system-level testing, the skin patches are simulated via FEA; therefore, their constitutive equations and material constants must be identified from additional mechanical tests, as described in Section 2.2. DragonSkin 30 is modeled as purely hyperelastic, an engineering approximation consistent with its lower energy dissipation ratio, whereas Ecoflex Gel-000-35 is treated as visco-hyperelastic. Figures 6a,b summarizes the visco-hyperelastic fitting results for DragonSkin 30 and Ecoflex Gel-000-35, respectively. Several hyperelastic models are fitted to the average quasi-static stress-strain response (red dots, averaged over three tests), namely, Neo-Hookean (blue), Ogden (green), and Yeoh (magenta). The Yeoh model provides the best fit for both materials, in agreement with the statistical analysis reported in (Ferrentino et al., 2021). In addition, Ecoflex Gel-000–35 specimens are characterized via dynamic frequency-sweep tests to identify their viscoelastic parameters. Figure 6c reports the resulting storage $\left(G^{\prime }\right)$ and loss $\left(G^{\prime \prime }\right)$ shear moduli as functions of strain frequency. The viscoelastic candidates include the Standard Linear Solid (SLS) model, commonly adopted for elastomers, with first- (blue) and second-order (green) Maxwell generalizations, and first (black) and second-order (magenta) generalized Kelvin-Voigt models. Based on these results, the second-order SLS-Maxwell model provides the best agreement with the experimental data. The material constants extracted from the fits for both materials are listed in Table 1 (both materials are assumed incompressible). Hence, as detailed in Section 2.1, several skins are benchmarked in quasi-static and impact conditions using the following configurations:

- Design D_1_-gel, employing only Ecoflex Gel-000-35.
- Design D_1_-dragon, employing only DragonSkin 30.
- Design D_2_, combining both materials, with DragonSkin 30 used for the support sleeve and Ecoflex Gel-000-35 used for the external skin layer, and it is the SkinAxis reference design.

**FIGURE 6:**
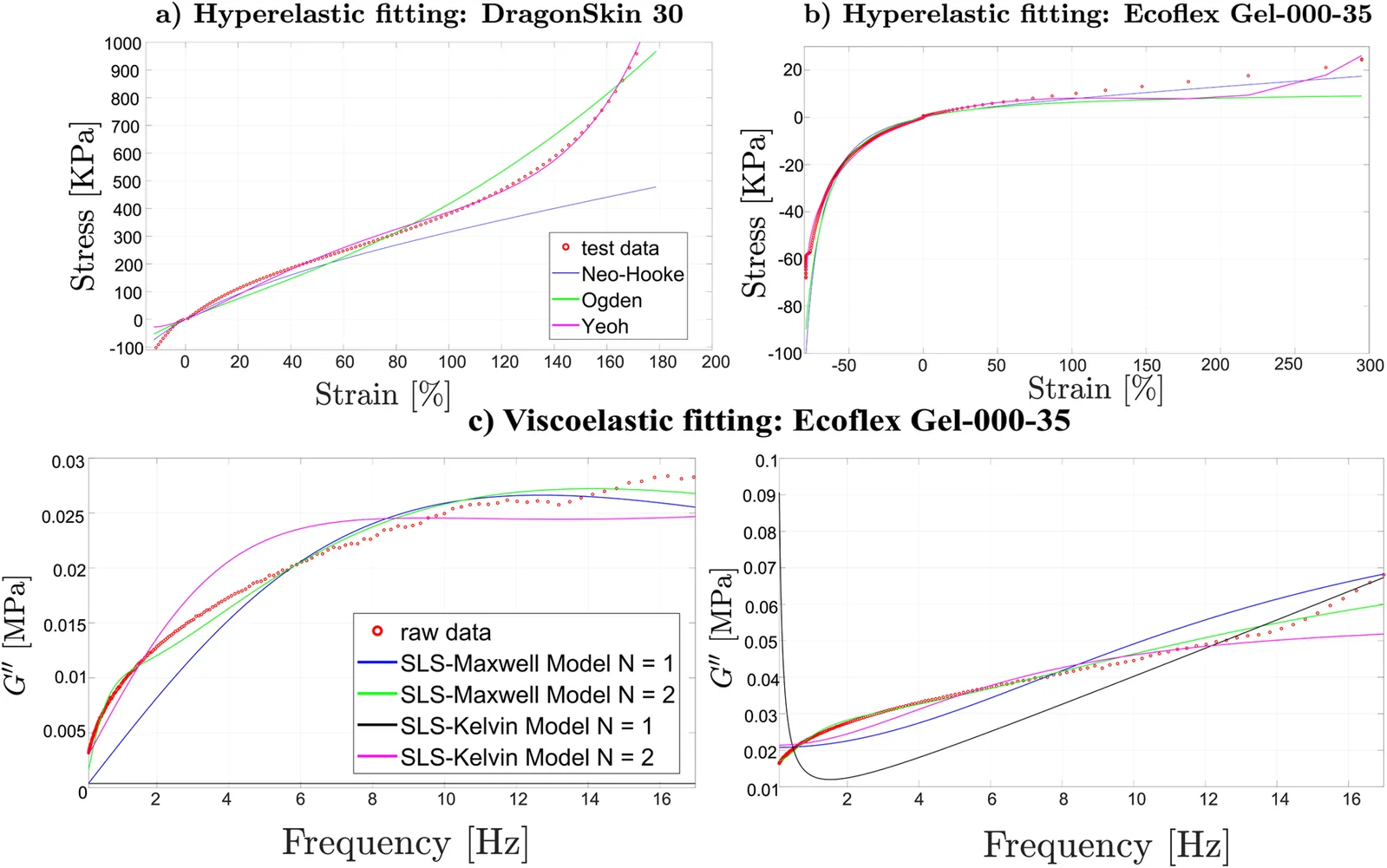
Results of the material characterization of DragonSkin 30 and Ecoflex Gel-000–35 materials. **(a)** The DragonSkin 30 is assumed to be purely hyperelastic due to its low energy dissipation ratio. Several hyperelastic equations are fitted to the quasi-static stress-strain curve (red dots). In particular, the Neo-Hooke (blue), Ogden (green), and Yeoh (magenta). The plot indicates that the Yeoh constitutive model is the best-fitting hyperelastic model. **(b)** The Ecoflex Gel-000-35 is assumed to be visco-hyperelastic; the quasi-static stress-strain curve undergoes the same fitting as the DragonSkin 30, and also in this case, the Yeoh constitutive model is the best-fitting. **(c)** The Ecoflex Gel-000–35 specimens are also subjected to a dynamic test in a frequency sweep to retrieve the loss and the storage modulus of the material as a function of the strain frequency. Several viscoelastic models are fitted to these curves, including SLS-Maxwell models of first (blue) and second (green) order and SLS-Kelvin-Voigt models of first (black) and second (magenta) order. The plot shows that the SLS-Maxwell second-order is the best fit.

**TABLE 1:** The constitutive equations and material constants for DragonSkin 30 and Ecoflex Gel-000-35. The elastic components are in MPa, while the viscous ones are represented by characteristic relaxation time constants (τ) , expressed in seconds.

	DragonSkin 30	Ecoflex gel 000-35
Constitutive equation	Yeoh	Yeoh + SLS-Maxwell N = 2
Material constants	${\text{C}}_{10}$ = 0.069 MPa, ${\text{C}}_{20}$ = 0.012 MPa, ${\text{C}}_{30}$ = 1.130 $\times 10^{-4}$ MPa	${\text{C}}_{10}$ = 0.003 MPa, ${\text{C}}_{20}$ = −2.825 $\times 10^{-4}$ MPa, ${\text{C}}_{30}$ = 1.455 $\times 10^{-5}$ MPa, ${\text{G}}_{1}$ = 0.013 MPa, $\tau_{1}$ = 0.166 s, ${\text{G}}_{2}$ = 0.053 MPa, $\tau_{2}$ = 0.011 s

### 3.2 Quasi-static force sensitivity results

In Figure 7a, the force-displacement curves for all three skins are shown: the FEA simulation (blue) and two experimental compressive tests on the Mark-10 (red and green). The two single-material baselines, made with only Ecoflex Gel 000-35 and only DragonSkin 30, represent, respectively, the most and least compliant designs, with maximum compressive displacements of 13 and 6 mm. Furthermore, they represent the softer and stiffer designs in the selection, with maximum input forces of 32 and 330 N, respectively. Instead, the design D_2_ represents the trade-off between compliance and stiffness of the two baselines, with a maximum displacement of 9.5 mm and a maximum compressive force of 100 N. For all three cases (D_1_-gel, D_1_-dragon, and D_2_), the FEA simulation is in agreement with the experimental results, having a Mean Absolute Error (MAE), averaged for all the experimental trials, of 3.75 % , 4.37 % and 2.23 % , and an averaged Root Mean Squared Error (RMSE) of 3.74 % , 3.77 % , and 3.13 % . The same MAE and RMSE values are reflected in Figure 7b, which plots the sensitivity curves for all the skin designs, using the same color palette as Figure 7a, demonstrating that the force-displacement model can well predict not only the overall force-displacement behavior but also the force transmitted to the encapsulated sensor inside the skin patches. From the trends of the sensitivity curves of Figure 7b, it is observable that in the skin D_1_-gel, the Tactaxis® sensor measures negligible forces, already announcing that this configuration is less sensitive. For the skin patches D_1_-dragon and D_2_ the force sensor readings increase quasi-linearly up to a maximum of 5 N, indicating that these configurations are more sensitive. According to the metrics defined in Section 2.3, the actual sensitivity levels ( ${\text{k}}_{\varepsilon }$ ) for all three patches are calculated, and illustrated them in Figure 7c in red (D_1_-gel), green (D_1_-dragon), and blue (D_2_), with values of 0.006 ± 0.001, 0.020 ± 0.005 and 0.089 ± 0.007, respectively. Hence, the SkinAxis reference configuration D_2_ is the most sensitive.

**FIGURE 7:**
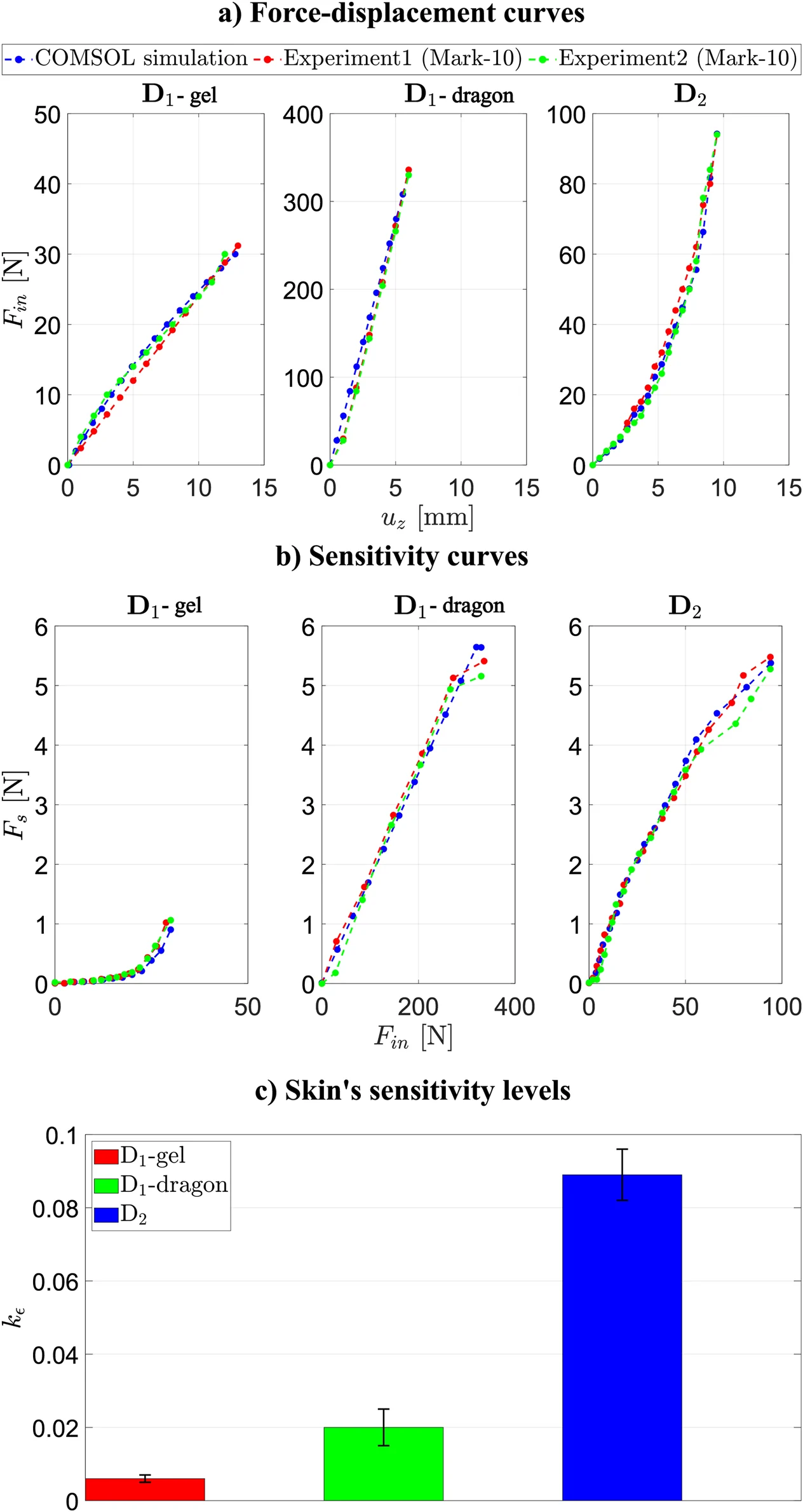
**(a)** Force-displacement curves for every skin configuration: D_1_-gel, D_1_-dragon and D_2_. The FEA simulation results are shown in blue; the experimental curves retrieved from the Mark-10 are shown in green and red. The configuration D_1_-gel is the most compliant but also the softer, while the configuration D_1_-dragon is the stiffer and the less compliant. The configuration D_2_ represents a trade-off between the two baseline configurations. **(b)** The sensitivity curves, in the same color palette as the previous image, show that the design D_1_-gel is the least sensitive one, while the other two have significant forces transmitted to the sensor. **(c)** The levels of sensitivity for every skin, in red D_1_-gel, green D_1_-dragon, and blue D_2_. The design D_2_ is the most sensitive. The values are reported as mean ± standard deviation, indicated as error bars.

### 3.3 Touch mapping results

Table 2 summarizes the mean localization errors in training and validation, $e_{\text{train}}$ and $e_{\text{val}}$ , for the best feature set of each regression model across the three skin designs D_1_-gel, D_1_-dragon, and D_2_. For D_1_-gel, Gaussian Process Regression with Matérn 5/2 kernel (GPR_M52) achieves the lowest validation error $\left(e_{\text{val}}\approx 2.3\text{ mm}\right)$ and is therefore selected as the preferred model. Other models on D_1_-gel exhibit similar training errors but slightly higher $e_{\text{val}}$ , indicating less accurate localization on unseen data. For D_1_-dragon, the best model is the BaggedTrees $\left(e_{\text{val}}≳6.7\text{ mm}\right)$ , but with a larger discrepancy between training and validation performance, suggesting that either the considered model classes are not expressive enough or that the sensor-derived force features are only weakly correlated with the surface contact location in this skin. This observation is consistent with the fact that D_1_-dragon uses a stiffer single-material architecture, which tends to concentrate loads less uniformly and may reduce the spatial information conveyed by the sensor readings. For D_2_, NN_2 × 10 provides the most favorable trade-off between accuracy and robustness, with $e_{\text{val}}\approx 4.3\text{ mm}$ , and is selected as the preferred model for subsequent analysis and real-time visualization. Several other models on D_2_ achieve comparable training errors but larger validation errors, confirming that NN_2 × 10 offers the best mapping from force features to contact location for the hybrid reference design.

**TABLE 2:** Mean training and validation localization errors (in mm) for the best feature set of each regression model and each skin design. For each skin, the model with the lowest validation error $e_{\text{val}}$ is selected as the preferred configuration for subsequent analysis (green). Training errors $e_{\text{train}}$ are reported for completeness to indicate the fit on the training data; large discrepancies between $e_{\text{train}}$ and $e_{\text{val}}$ suggest limited generalization for some models.

Model	D_1_-gel		D_1_-dragon		D_2_	
	$e_{\text{train}}$ [mm]	$e_{\text{val}}$ [mm]	$e_{\text{train}}$ [mm]	$e_{\text{val}}$ [mm]	$e_{\text{train}}$ [mm]	$e_{\text{val}}$ [mm]
GPR_M52	**1.6**	**2.3**	4.07	8.9	3.0	4.6
GPR_SE	1.8	2.5	4.5	9.1	3.1	4.4
NN_2 × 10	3.0	3.0	9.6	10.3	**4.0**	**4.3**
NN_2 × 20	3.2	3.1	10.3	10.9	4.0	5.0
NN_1 × 20	3.5	3.3	10.2	11.7	4.1	5.1
NN_1 × 10	3.8	3.3	13.0	13.2	4.5	5.4
BaggedTrees	1.0	2.8	**2.4**	**6.7**	1.5	5.4
LSBoostTrees	1.0	2.9	4.7	7.9	1.5	4.9
SVM_Gaussian	4.6	4.1	9.4	11.1	7.4	9.1

The performance of the selected models also depends on the indentation force level $F_{\text{in}}$ used during data collection. The force ranges were empirically chosen so that, over the entire top surface of each patch, the embedded Tactaxis® sensor produced substantial normal and shear readings (at least 0.25 N) when indenting multiple locations on the surface. For D_1_-gel and D_2_, the range of indentation forces used was 2–5 N (with 1 N step), while for D_1_-dragon it was 10–40 N (with 10 N step), as DragonSkin 30 is stiffer and need more force to be deformed by the indenter. Hence, the determination of the parameter $S_{\text{touch}}$ is conducted only on the skin patches D_1_-gel and D_2_, because they work in the same ranges of forces. The results are listed in Table 3, where $F_{\min}$ is 2 N and $S_{\text{touch}}$ takes values of 2.3 and 2.7 mm $N^{-1}$ for D_1_-gel and D_2_, respectively. This indicates that D_1_-gel yields a lower localization error per unit force at its operating level, but its selected model exhibits a strong discrepancy between training and validation errors, which persists when increasing $F_{\min}$ in the 2–5 N range and suggests limited robustness of the learned mapping. In contrast, the model for D_2_ remains consistent across force levels and provides a more reliable mapping, making D_2_ more attractive for practical deployment despite its larger $S_{\text{touch}}$ . For this reason, the patch D_2_ and its trained/validated model are used in the real-time demonstration, where the estimated touch location is visualized on an RGB map of the skin’s top surface whenever the indenter contacts the skin, as shown in Figure 8 and in the video accompanying this work.

**TABLE 3:** Localization performance of the selected models at specific indentation force levels, with touch sensitivity defined as $S_{\text{touch}}=e_{\text{mean}}\left(F_{\text{in}},D_{\text{val}}\right)/F_{\min}$ on the corresponding validation subset.

Skin	$F_{\min}$ [N]	$e_{\text{train}}$ [mm]	$e_{\text{val}}$ [mm]	$S_{\text{touch}}$ [mm $N^{-1}$ ]	Notes
D_2_	2	5.8	5.4	2.7	Stable validation error
D_1_-gel	2	0.3	4.7	2.3	Large train–val discrepancy

**FIGURE 8:**
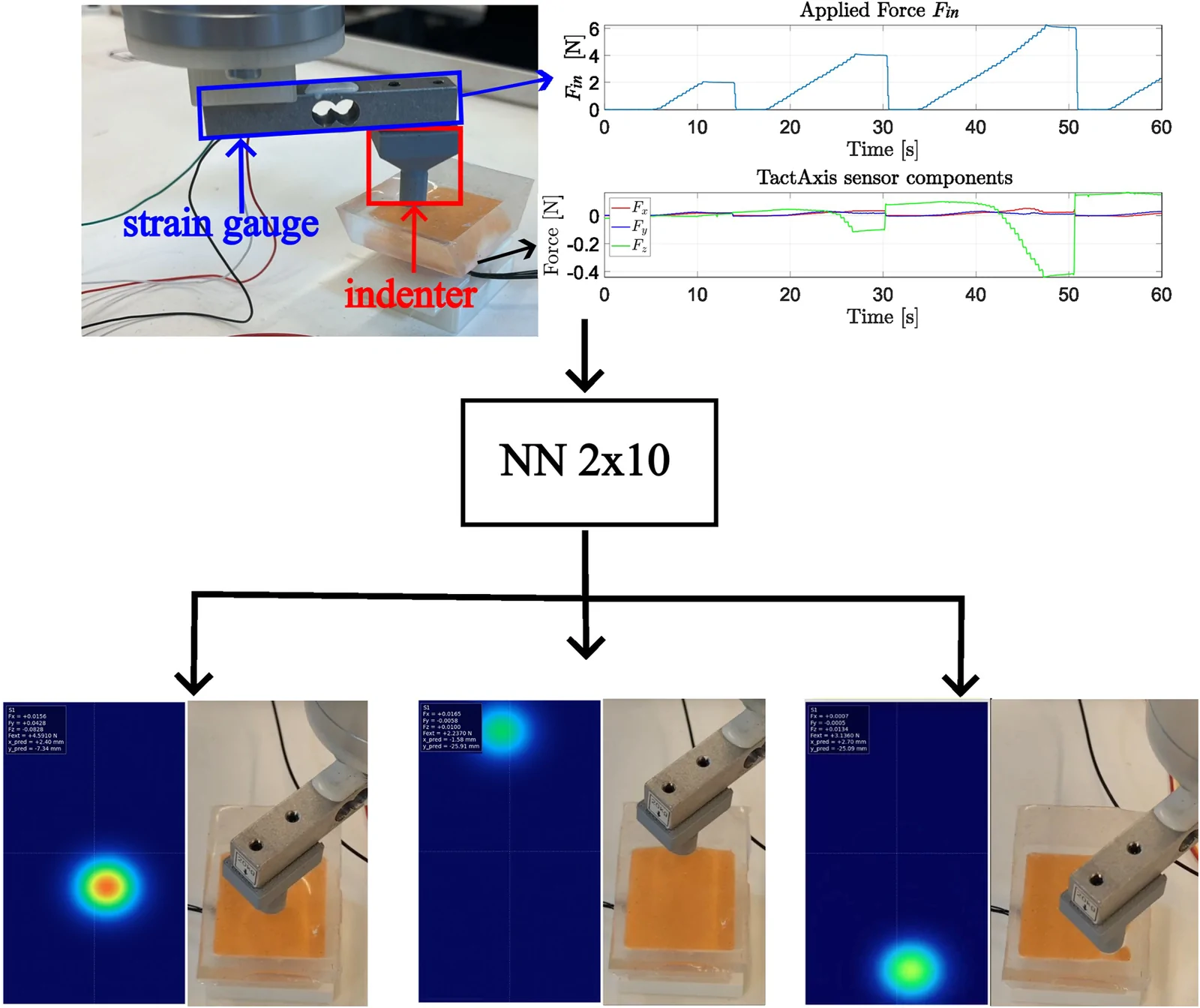
A neural network with two hidden layers of 10 neurons each takes in input the indentation force and the readings of the Tactaxis® sensor, and gives in real-time as output the (x,y) position, enclosed in the indenter area, on an RGB map of the top surface.

### 3.4 Impact tests and simulation results

In Figures 9a–d, the impact forces recorded by the PILZ PRMS setup are shown. Figure 9a reports the case in which no skin is involved in the collision (baseline), whereas Figures 9b–d corresponds to the designs D_1_-gel, D_1_-dragon, and D_2_, respectively. The impacts are performed at three different cobot speeds: 0.2 $\frac{m}{s}$ (red), 0.6 $\frac{m}{s}$ (green), and 1 $\frac{m}{s}$ (blue). All collision forces are averaged over three repeated trials at the same speed. The Figure highlights how the cobot’s operating speed affects impact forces during accidental collisions, with peak forces increasing markedly at higher velocities. Comparing the baseline with the benchmarked cases, it is observed that when the patches are involved in the collision, the impact force profiles are spread over time (particularly evident at $v_{\text{cobot}}=0.2\;\frac{m}{s}$ ), and the peak forces are reduced due to the damping effects of the skins. It can already be noticed that the patch D_1_-dragon provides limited peak force reduction, as DragonSkin 30 is a highly elastic elastomer with low dissipation, whereas D_1_-gel and D_2_ exhibit substantially better impact force attenuation.

**FIGURE 9:**
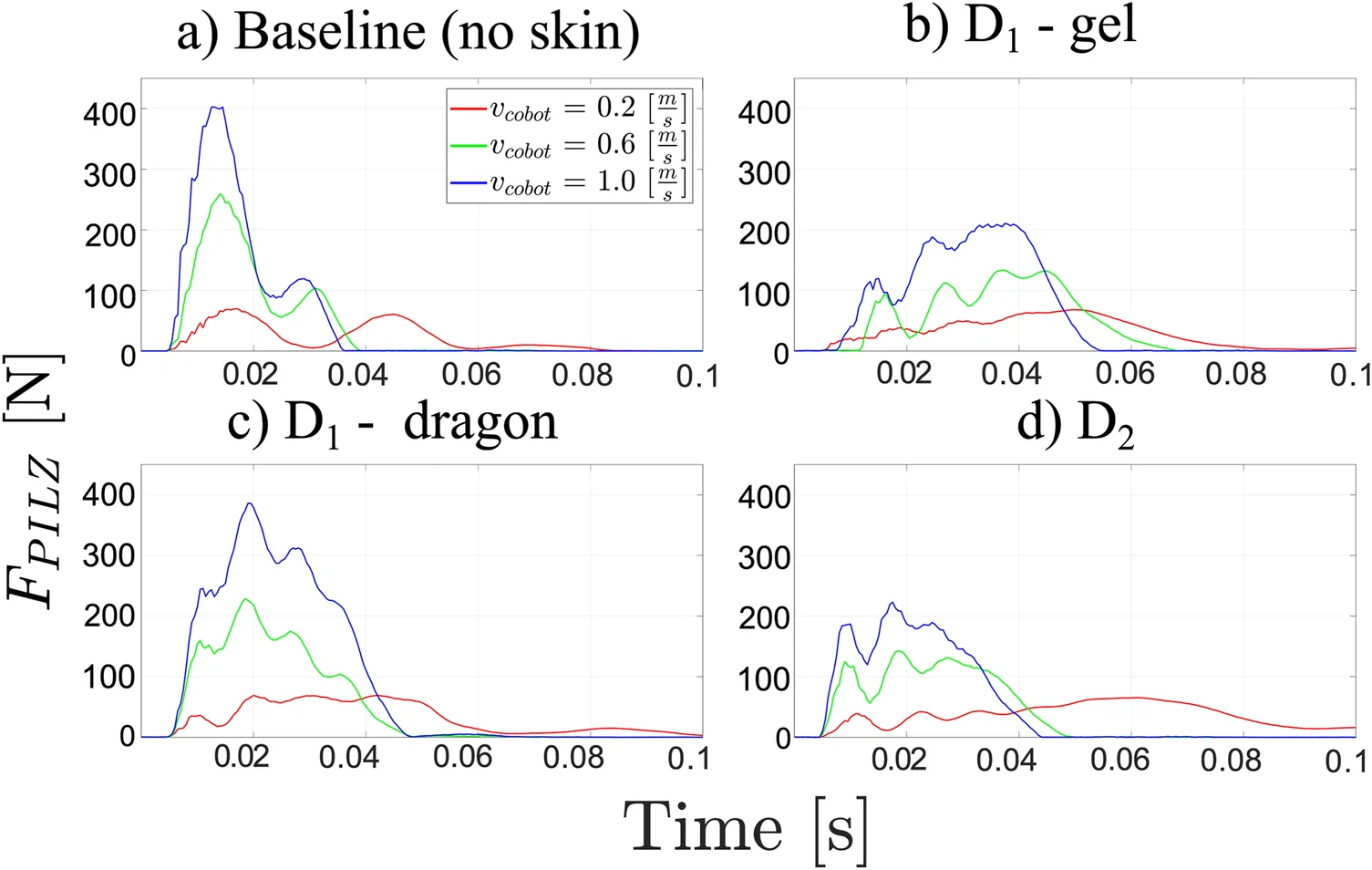
This Figure illustrates the impact forces over time recorded from the PILZ PRMS setup $\left(F_{\mathrm{PILZ}}\right)$ for the baseline (no skin) impact **(a)**, the skin patch D_1_-gel **(b)**, D_1_-dragon **(c)**, and reference SkinAxis design D_2_ **(d)**. The impact tests were conducted at three different cobot velocities: 0.2 $\frac{m}{s}$ (red), 0.6 $\frac{m}{s}$ (green), and 1 $\frac{m}{s}$ (blue). When the skin is involved in the impact, the collision forces are spread over time, offering a damping effect. While skin D_1_-dragon does not exhibit a significant reduction in peak force, D_1_-gel and D_2_ show substantially lower peak forces at the same prescribed operational velocity.

At $v_{\text{cobot}}=0.2$ m/s, the estimated average nominal strain rates were 3.63 ${\text{s}}^{-1}$ for D_1_-gel, 2.42 ${\text{s}}^{-1}$ for D_1_-dragon, and 2.23 ${\text{s}}^{-1}$ for D_2_. At $v_{\text{cobot}}=0.6$ m/s, the corresponding values increased to 10.56 ${\text{s}}^{-1}$ , 11.39 ${\text{s}}^{-1}$ , and 12.33 ${\text{s}}^{-1}$ , respectively. At the maximum tested velocity of $v_{\text{cobot}}=1$ m/s, the estimated strain rates were 15.06 ${\text{s}}^{-1}$ for D_1_-gel, 12.82 ${\text{s}}^{-1}$ for D_1_-dragon, and 15.74 ${\text{s}}^{-1}$ for D_2_. Although the nominal strain rates are thus comparable across the three patches, particularly at 1 m/s, the impact experiments show substantially larger peak-force and transmitted-energy reductions for the gel-based D_1_-gel and hybrid D_2_ patches than for the single-material D_1_-dragon patch. This observation is consistent with the low dissipative contribution of DragonSkin 30 identified during material classification and supports its use as a hyperelastic first-order engineering approximation in the present system-level impact simulations.

To quantify these effects, the relative peak force reduction efficiency $\eta_{F}^{\left(-\right)}$ and the relative energy transmission reduction efficiency $\eta_{E}^{\left(-\right)}$ for each skin patch and cobot speed are reported in Table 4. The same table also lists the maximum force and maximum pressure at $v_{\text{cobot}}=1\;\frac{m}{s}$ . The maximum pressure indicates whether a given patch meets the ISO/TS 15066 safety condition for maximum collision pressure on a human head ( ≤1.30 MPa). The table confirms that the designs D_1_-gel and D_2_ achieve the largest peak force reduction, with $\eta_{F}^{\left(-\right)}$ values of [2.56%, 48.47%, 44.79%] for D_1_-gel and [6.53%, 45.34%, 44.66%] for D_2_. At 0.2 $\frac{m}{s}$ , the efficiency is lower due to reduced squeezing compression of the skin against the PILZ setup. These patches also provide the highest reduction in energy transmission to the PILZ, namely, [44.79%, 35.94%, 31.60%] for D_1_-gel and [43.30%, 35.93%, 18.95%] for D_2_. Furthermore, at $v_{\text{cobot}}=1\;\frac{m}{s}$ , the maximum pressures are 1.05 MPa and 1.11 MPa for the two designs, respectively, ensuring compliance with ISO/TS 15066 requirements at typical cobot operating speeds.

**TABLE 4:** This table reports the relative efficiency metrics for the benchmarked skins at different prescribed operational velocities, together with the maximum force and pressure (in MPa) for $v_{\text{cobot}}=1\;\frac{m}{s}$ . The designs D_1_-gel and D_2_ provide the largest reduction in peak force and transmitted energy and remain safe, in terms of maximum pressure applied on the PILZ at $1\;\frac{m}{s}$ , with respect to the ISO/TS 15066 limits. Quantities are reported as mean ± standard deviation.

Skin patch	$\eta_{F}^{\left(-\right)}$ [%]			$\eta_{E}^{\left(-\right)}$ [%]			$F^{\max}$ [N]	$P^{\max}$ [MPa]
	**0.2** $\frac{m}{s}$	**0.6** $\frac{m}{s}$	**1.0** $\frac{m}{s}$	**0.2** $\frac{m}{s}$	**0.6** $\frac{m}{s}$	**1.0** $\frac{m}{s}$		
D_1_-gel	2.56±0.23	48.47±3.24	45.68±2.96	44.79±2.66	35.94±1.78	31.60±2.43	210.62±2.84	1.05±0.02
D_1_-dragon	0.93±0.05	11.76±1.45	4.09±1.20	35.22±3.23	29.88±2.34	26.22±2.52	386.16±5.89	1.93±0.03
D_2_	6.53±0.56	45.34±2.76	44.66±2.45	43.30±3.48	35.93±2.36	18.95±1.25	222.82±3.19	1.11±0.02

In Figures 10A–C, the results of the simulation and experimental validation of the Tactaxis® force norm $F_{s}$ under impact conditions are reported for each design, using the Gaussian pulse approximation for the impact displacement as described in Section 2.5. Each column of Figure 10 corresponds to one of the three tested prescribed operational velocities (0.2, 0.6, and 1 m/s). The simulation curves (shown in blue) are compared with experimental Tactaxis® measurements obtained from three repeated impacts at the same cobot velocity (reported in red, orange, and magenta). Across all designs and velocities, the measured force norms closely follow the simulated profiles, confirming that the Gaussian pulse approximation for the impact displacement provides a good dynamic validation of the sensor response, as also demonstrated by the MAE and RMSE values (averaged over the three trials) reported in Table 5. The nonlinear time-dependent solver was terminated using a relative tolerance of 0.1, while PARDISO was employed as the direct linear solver within the nonlinear iterations. This setting was selected to achieve robust convergence of the large-deformation contact simulations. A solver-sensitivity analysis using the tightest convergent tolerance settings, such as 0.05 and 0.02, confirmed that the resulting changes in the predicted sensor force were about 2 % , smaller than the corresponding simulation–experiment error.

**FIGURE 10:**
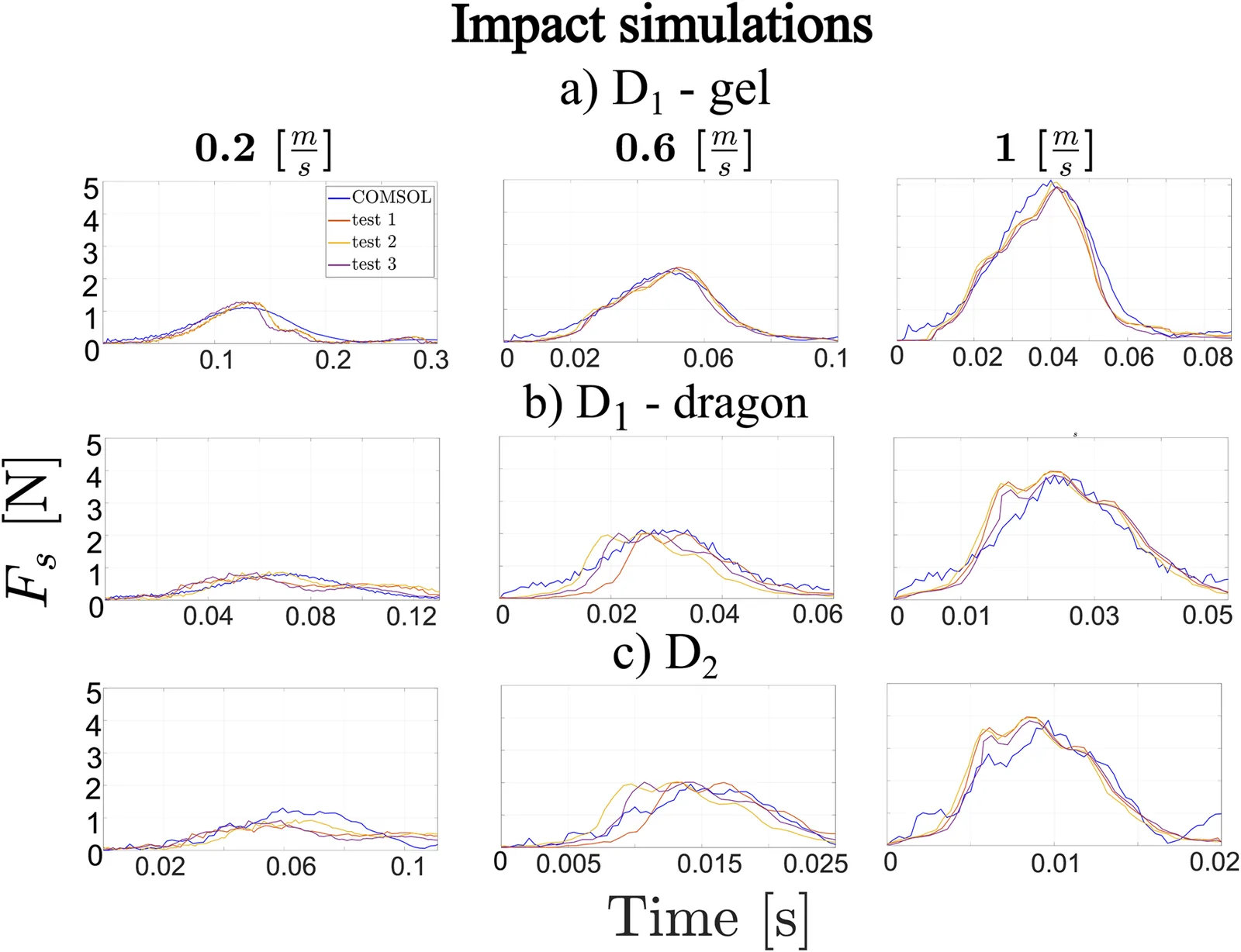
**(a-c)** Simulation validation results of the force norm $F_{s}$ of the Tactaxis® sensor in Impact conditions. The results are relative D_1_-gel, D_1_-dragon and D_2_ design configurations. The three columns represent the different prescribed operational velocities (0.2, 0.6, and 1 $\frac{m}{s}$ ). The FEA simulation results are reported in blue, while the experimental results for the Tactaxis® sensor under three different impacts are shown in red, orange, and magenta at the same cobot’s velocity. The plots confirm that the Gaussian pulse approximation of the impact displacement gives good dynamic validation results.

**TABLE 5:** Mean Absolute Error (MAE) and Root Mean Square Error (RMSE) between simulated and measured Tactaxis® force norm $F_{s}$ under impact conditions, expressed in percent and averaged over three trials for each SkinAxis design and prescribed operational velocity.

Velocity [m/s]	$D_{1}-gel$		$D_{1}-dragon$		$D_{2}$	
	MAE [%]	RMSE [%]	MAE [%]	RMSE [%]	MAE [%]	RMSE [%]
0.2	8.50	8.74	8.65	8.88	9.44	9.56
0.6	8.36	8.43	10.44	10.37	10.50	9.88
1.0	9.24	9.14	10.63	10.89	11.25	11.06

## 4 Discussion, conclusion, and future work

The results highlight that the reference SkinAxis patch D_2_ offers the most favorable trade-off between the two single-material baselines, the soft dissipative D_1_-gel and rigid D_1_-dragon, directly reflecting the material-based design strategy adopted in this work. The overall aim of the paper is to design soft skins that transmit sufficient force to an embedded 3D sensor for localized touch perception, while still absorbing and mitigating impact loads in accordance with ISO/TS 15066. This dual objective is addressed by selecting and combining elastomers with complementary mechanical properties: a stiff, high-AED material (DragonSkin 30) that enhances load transmission to the sensor, and a highly dissipative gel (Ecoflex Gel-000–35) that attenuates impact forces and energy.

In quasi-static compression, D_2_ achieves the highest normalized sensitivity $k_{\varepsilon }$ , indicating that it transmits more force to the embedded 3D sensor per unit of applied load while remaining compliant and comfortable under touch. This outcome is consistent with its hybrid architecture: the stiff DragonSkin sleeve and PLA plate concentrate and channel deformations towards the sensor, whereas the softer gel encapsulation preserves surface compliance. In contrast, the fully dissipative single-material baseline D_1_-gel, which uses only the highly dissipative elastomer, exhibits very low $k_{\varepsilon }$ despite its cushioning properties, confirming that impact-oriented materials alone are insufficient to provide strong quasi-static force sensitivity.

In terms of touch sensitivity and localization, D_1_-gel attains the lowest $S_{\text{touch}}$ value, i.e., the smallest localization error per unit force at its operating level, but the corresponding model shows a strong discrepancy between training and validation errors and remains sensitive to the specific force range used for training. This behavior reflects the fact that the dissipative gel can make certain touches effectively “invisible” or highly non-uniform at the sensor as forces vary, thereby limiting the robustness of the learned mapping. In contrast, the hybrid reference design D_2_ provides more consistent localization at lower $F_{\min}$ , with moderate validation errors over a wider force range, making it more practical for deployment on collaborative robots, where touch events span a broader spectrum of contact forces. Here, the presence of the stiff sleeve and plate ensures that small variations in contact force are reliably transmitted to the sensor, while the gel layer still contributes to comfort and impact safety.

Under impact conditions, D_1_-dragon proves insufficient to guarantee safe physical human–robot collaboration at 1 m/s, despite its excellent load-transmission capacity, because the stiff DragonSkin material has low dissipation and transmits high peak forces and energy. The fully dissipative baseline D_1_-gel, instead, markedly reduces peak forces and transmitted energy and keeps maximum pressures within ISO/TS 15066 limits, but at the cost of reduced quasi-static sensitivity and less robust touch localization. The reference SkinAxis design D_2_ stands out as a balanced solution: it preserves high quasi-static sensitivity and usable touch localization while providing substantial impact mitigation in a compact, easy-to-fabricate architecture, demonstrating that a material-based combination of stiff and dissipative elastomers can simultaneously support sensing and safety. Also, the dynamic FEA model used a prescribed displacement boundary condition, in which the experimentally observed patch-compression history was represented by a fitted Gaussian-shaped pulse. It was therefore intended to reproduce the sensor-level force response of the patch under the tested kinematic input, rather than to predict the complete coupled robot–skin–human collision from robot commands. The model does not include robot configuration-dependent effective mass, robot-controller dynamics, end-effector and patch inertia, or a fully coupled biomechanical model of the PILZ head surrogate. Nevertheless, the simulated Tactaxis® force norms agreed satisfactorily with the experimental signals.

Overall, the study shows that modular soft robotic skins can jointly provide localized 3D force sensing and impact protection by explicitly exploiting material properties rather than relying on a single compromise material. The comparison between D_1_-gel, D_1_-dragon, and the hybrid reference SkinAxis patch D_2_ quantifies how combining high-AED, load-transmitting elastomers with highly dissipative gels around a compact force sensor affects quasi-static sensitivity, touch localization performance, and impact safety. Among the tested designs, D_2_ emerges as the most balanced option, offering high quasi-static sensitivity, practical touch localization, and impact safety compliant with ISO/TS 15066 at 1 m/s.

Future work will focus on extending the reference SkinAxis design to multi-patch configurations across larger robot surfaces, integrating additional sensing modalities (such as proximity or temperature), and incorporating varied contact indentation geometries with multiple loading directions to improve and generalize touch localization. In addition, it will be assessed whether the mount is durable and whether the design experiences long-term material aging. Furthermore, closed-loop control strategies that exploit SkinAxis measurements to enable real-time adjustment of cobot motion and compliance during physical human–robot collaboration can be created.

The impact experiments considered only a centered, nominally normal contact configuration. All baseline and skin-covered trials were conducted under the same nominal robot posture, impact trajectory and direction, controller settings, mounting interface, and PILZ PRMS configuration. Although the added skin mass may slightly increase the effective inertia of the end-effector assembly, this effect cannot account for the measured reductions in peak force and transmitted energy at a prescribed operational velocity. The results therefore support the impact-mitigation role of the skin material and patch architecture. Future work should quantify how patch placement, edge effects, off-center contact, and oblique impacts affect impact mitigation when the skins are deployed on curved robot links, and also quantify the direction-dependent effective mass at the actual contact point using recorded robot joint states and an inertial model of the complete assembly. Also, in the future, a fully coupled finite element model will be developed to predict collision outcomes across robot configurations, contact geometries, and human-body surrogates.

In parallel, the material-based methodology can be generalized to other elastomer families and cellular architectures, enabling the systematic design of robotic skins in which material selection and arrangement are tuned to achieve application-specific trade-offs between touch sensitivity, impact mitigation, and mechanical comfort. Beyond collaborative arms, these developments will also be explored on humanoids and quadrupeds, where modular protective skins can mitigate forces and pressures arising from falls and contacts during locomotion over uneven or unstructured terrain.

## Data Availability

The raw data supporting the conclusions of this article will be made available by the authors, without undue reservation.
